# Impact of Symptom Distress on the Quality of Life of Oncology Palliative Care Patients: A Portuguese Cross-Sectional Study

**DOI:** 10.3390/healthcare12232487

**Published:** 2024-12-09

**Authors:** Florbela Gonçalves, Margarida Gaudêncio, Ivo Paiva, Valéria Andrade Semedo, Francisca Rego, Rui Nunes

**Affiliations:** 1 Portuguese Institute of Oncology Francisco Gentil Coimbra, 3000-075 Coimbra, Portugal; 4196@ipocoimbra.min-saude.pt; 2 Faculty of Medicine, University of Porto, 4099-002 Porto, Portugal; mfrego@med.up.pt (F.R.); ruinunes@med.up.pt (R.N.); 3 Health Sciences Research Unit, Nursing, Nursing School of Coimbra, 3000-232 Coimbra, Portugal; ivopaiva@esenfc.pt; 4 Universitary Hospital Dr. Agostinho Neto, Praia 7600, Cape Verde; valeria.semedo@huan.gov.cv

**Keywords:** hospice, palliative care, palliative medicine, patient care, quality of healthcare, quality of life, symptom assessment

## Abstract

Introduction: Uncontrolled symptoms are widely recognized as one of the main challenges in oncology palliative care patients. The central aim of palliative care is to improve the patient’s quality of life. In recent years, there has been a growing use of patient-reported outcome measures in palliative care, particularly to evaluate symptoms, quality of care, and well-being. Aim: To evaluate the sociodemographic and clinical profile, symptom distress, and perceived quality of life in oncology palliative care patients admitted to a specialized palliative care unit in Portugal. Methods: This study was cross-sectional, descriptive, and correlational, carried out in the inpatient setting of the palliative care unit at a tertiary oncology hospital (at admission). The evaluated protocol included a sociodemographic and clinical questionnaire, as well as two measurement instruments: the Edmonton Symptom Assessment Scale (ESAS) and the Palliative Care Outcome Scale (POS), both filled out by the patients. Data analysis was conducted using IBM SPSS^®^ Statistics version 25.0, with a significance level set at 5% (*p* < 0.05). Results: The majority of participants in this sample were male (61.7%), with a mean age of around 72 years. More than half of the patients admitted (n = 34; 56.7%) were being monitored in outpatient care. Digestive and head and neck cancers were the most commonly found in the sample (41.7% and 20%, respectively). A significant correlation was found between high symptom intensity and poorer quality of life and care (*p* < 0.01). This association was particularly pronounced for symptoms such as pain, weakness, depression, anxiety, and anorexia. Conclusions: This study revealed a positive correlation between overall symptom severity and a perceived deterioration in quality of life, well-being, and quality of care. Future studies should consider utilizing alternative assessment tools for evaluating symptoms and quality of care. Additionally, including non-cancer palliative patients in similar studies may provide further valuable insights.

## 1. Introduction 

Cancer represents a significant global public health issue. Both cancer morbidity and mortality are on the rise worldwide, with an estimated 16 million global cancer-related deaths projected by 2024 [1].

Palliative care for cancer patients should be initiated at the onset of the disease and continue through to the bereavement period, prioritizing quality of life and achieving optimal symptomatic control [2,3]. 

Palliative care is a holistic and humanized approach that aims to improve the quality of life for patients and families facing life-threatening illnesses [4,5]. Patients with highly complex symptoms are the ones who benefit most from specialized palliative care. It is well established that cancer patients generally experience a lower quality of life compared to the general population [6,7].

The literature demonstrates that the presence of uncontrolled symptoms has a detrimental effect on the quality of life and overall satisfaction of these patients [8,9]. Therefore, the essential components of symptom management are accurate identification, thorough evaluation, and appropriate treatment [10]. To ensure a good quality of life, it is essential to document the effectiveness of interventions carried out to reduce physical and psychological suffering [2]. Furthermore, it is also necessary to evaluate and monitor these interventions over time [2].

Several assessment and measurement instruments have been developed to evaluate symptom distress [9]. Some multidimensional questionnaires are overly complex for debilitated patients, while others, such as the visual analog scale and numerical rating scale, are simple and highly effective [9].

According to the World Health Organization (WHO), quality of life is defined as “an individual’s perception of their position in life, within the context of the culture and value systems in which they live, and in relation to their goals, expectations, standards, and concerns” [11].

Measuring the quality of life of cancer patients at various stages of their illness is considered a fundamental clinical practice [12]. In the past, indicators of the success or failure of therapeutic interventions were largely confined to response rates, disease-free survival intervals, or mortality rates [12].

In 1996, the American Society of Clinical Oncology (ASCO) adopted clinical guidelines for metastatic cancers, stating that treatment may be recommended based on its potential to improve quality of life, even in the absence of survival benefits [13].

Higginson and Carr proposed that quality of life measures serve several clinical purposes, including identifying problems, enhancing communication skills, screening for hidden issues, facilitating shared decision-making, and monitoring health changes or treatment effects [14]. 

In 1949, Karnofsky et al. introduced the first instrument to assess functional levels in cancer patients [15]. This performance scale ranged from 0 (indicating death) to 100 (indicating complete autonomy) [15]. Subsequently, in 1982, the Eastern Cooperative Oncology Group (ECOG) developed a similar scale known as the Performance Status Scale to assess functional levels [16], along with the Quality of Life Questionnaire (QLQ-C30) from the European Organization for Research and Treatment of Cancer (EORTC) [17].

Measuring outcomes in palliative care requires the assessment of more specific dimensions, such as symptom control, improvement of quality of life before death, family support, and the satisfaction and meaning of life.

The Edmonton Symptom Assessment Scale (ESAS) is a multidimensional instrument developed by Bruera in 1991 at the palliative care unit of Edmonton General Hospital in Canada [18]. This tool is designed to track and measure the most common symptoms experienced in palliative care, both physical and psychological [18]. It evaluates ten symptoms in total: six related to the physical dimension (pain, fatigue, nausea, drowsiness, dyspnea, and appetite), two addressing the psychological dimension (depression and anxiety), one assessing overall well-being, and an optional symptom [18]. 

In this investigation, the Global Distress Scale (GDS) was used. This scale is a subtype of the Edmonton Symptom Assessment Scale (ESAS), designed to assess the presence and intensity of the first nine symptoms identified in the ESAS [19]. 

Additionally, the authors employed the Palliative Outcome Scale (POS) (patient self-report) to evaluate the well-being and quality of life of these individuals, as its questions and dimensions are well-suited for a population composed of cancer patients in palliative care [15].

The Palliative Outcome Scale (POS) was originally developed to assess the quality of care and well-being in palliative care patients [20,21]. Due to its multidimensional nature, it also serves as a measure of quality of life in palliative care, encompassing physical, psychological, emotional, and spiritual aspects of patients [12,22,23].

The authors recognize that the Edmonton Symptom Assessment Scale (ESAS), specifically the Global Distress Scale (GDS), is now commonly used to assess symptom intensity in patients undergoing palliative care [24]. The ESAS can be considered an instrument for evaluating quality of life, as it identifies physical and emotional symptoms and includes a nonspecific general item that assesses “well-being.” However, it does not assess the social and spiritual dimensions of quality of life in detail [24]. 

Although the Palliative Outcome Scale (POS) was originally designed as a tool to measure the quality of care and psychological well-being, several studies have utilized it to evaluate the quality of life of patients and their families in palliative care. This is due to the fact that its questions facilitate the assessment of physical, psychological, social, and spiritual dimensions [12,22,23]. 

So, with this study, the authors intend to analyze the sociodemographic and clinical profile of oncology palliative care patients admitted to a specialized palliative care unit in Portugal. Additionally, the study will evaluate symptom intensity and the perception of quality of life at admission, utilizing the Edmonton Symptom Assessment Scale (ESAS) and the Palliative Outcome Scale (POS) as measurement instruments. 

## 2. Material and Methods

### 2.1. Sample and Design

This was a cross-sectional, descriptive, and correlational study conducted in the palliative care unit of a Portuguese oncology hospital. The study included a sample of 60 consecutive patients admitted to the unit for the first time. 

The inclusion criteria were adult cancer patients undergoing palliative/supportive treatment at the hospital (who have suspended chemotherapy and radiotherapy), admitted to the palliative care ward between January and March 2021, who provided informed consent to participate and were able to comprehend the study’s objectives.

The authors excluded all patients under 18 years of age, as well as those who did not speak Portuguese, had communication difficulties, were terminally ill, or were unable to comprehend the study and/or provide written consent.

### 2.2. Ethical Approval, Informed Consent and Data Collection

The procedures followed were in accordance with the regulations of the Helsinki Protocol [25] and the Oviedo Convention [26] and obtained approval from the institution’s Ethics Committee (Opinion No. TI 17/2020).

All participants were provided with two sealed envelopes. One envelope contained a letter explaining the research and the informed consent form for them to sign, while the second envelope contained the measurement instruments. 

Data confidentiality was guaranteed through the separate collection of sealed envelopes, having been assigned an alphanumeric code ordered by registration in the database created for this purpose. The data collection period took place between January and March 2021. 

### 2.3. Social and Clinical Demographic Assessment and Measuring Instruments

The sociodemographic and clinical questionnaire, along with the measurement instruments, was provided to participants within the first 24 h of their admission to the palliative care ward and was completed by the participants themselves.

The sociodemographic and clinical questionnaire included variables such as age, sex, place of origin, type of neoplasm, and Eastern Cooperative Oncology Group performance status (ECOG). The ECOG scale (Boston, USA) assesses the functional status of patients, with scores ranging from 0 (fully active) to 4 (completely unable to perform self-care activities) [16]. 

Two measurement instruments were used to assess the patients in this study: the Edmonton Symptom Assessment Scale (ESAS), translated into Portuguese, aimed at identifying and rating the severity of the most common symptoms in palliative care, and the Palliative Care Outcome Scale (POS), also in its Portuguese version, which measures quality of life and well-being.

The Edmonton Symptom Assessment Scale (ESAS) evaluates ten symptoms in total: six related to the physical dimension (pain, fatigue, nausea, drowsiness, dyspnea, and appetite), two addressing the psychological dimension (depression and anxiety), one assessing overall well-being, and an optional symptom [18]. The ESAS allows patients to self-report the severity of their symptoms in their daily routine. It is a numerical scale ranging from 0 (absence of the symptom) to 10 (maximum possible intensity) [27]. 

The authors chose to use the Global Distress Scale (GDS), which consists of the first nine symptoms from the ESAS. Studies indicate that the GDS is correlated with patient survival rates [19]. The GDS was categorized into three levels: high (GDS ≥ 35), moderate (16–34), and low (0–15) [19]. 

The Palliative Outcome Scale (POS) consists of 11 questions scored on a Likert scale from 0 to 4, with both numerical and descriptive labels [21,28]. It is considered a valuable tool for assessing quality of life, as it addresses not only practical and psychosocial needs but also takes into account the patient–family dynamic as a whole [12,22,23]. The total POS score is calculated from the first 10 questions, ranging from 0 to 40 points, with higher scores indicating poorer quality of life and care [29].

### 2.4. Statistical Analysis

SPSS version 25 (IBM; Chicago; USA) was employed for statistical analysis. 

Each variable was characterized using the most appropriate statistical tests. Absolute and relative frequencies (N and %) were used to describe categorical and qualitative variables, while means, minimum, maximum, and quartiles were used for continuous variables. 

The authors performed Kruskal–Wallis tests to assess possible associations between demographic (gender, education level, marital status, number of children, companion and residence) or disease-related characteristics (type of neoplasm, ECOG) with ESAS and POS total score. 

Spearman’s coefficient was applied to assess correlations between variables (ECOG and POS score). 

A *p*-value of <0.05 was considered statistically significant for all comparisons.

## 3. Results

### 3.1. Sociodemographic Characteristics and Disease-Related Data of the Sample

During the 3-month study period, 60 patients were included for analysis. None of the patients declined participation and no other participants were excluded. 

In the study sample, the prevalence of men was higher than that of women (n = 37; 61.7% vs. n = 23; 38.3%). The average age of participants was approximately 72 years. Most patients were married and had one or two children. Eighty-three percent were living at home. Most patients were accompanied by their family members (spouse and/or children). Most patients had elementary education (81.7%). Epidemiological and social characteristics are further detailed in Table 1. 

More than half of the patients (n = 34; 56.7%) were referred from the external palliative care consultation, while the remaining patients (n = 26; 43.3%) were transferred from other services within the institution where they had been hospitalized. Regarding the type of neoplasm, the most frequently observed were digestive cancers, followed by head and neck cancers (41.7% and 20%, respectively) (Table 2).

Approximately one-third of the cases had an ECOG performance status of 3 or 4 upon admission (Table 2). 

On the Global Distress Scale (GDS), the median score was 42.5, with a minimum of 15 and a maximum of 62 (Table 2).

### 3.2. Edmonton Symptom Assessment Scale Results

**Figure 1:**
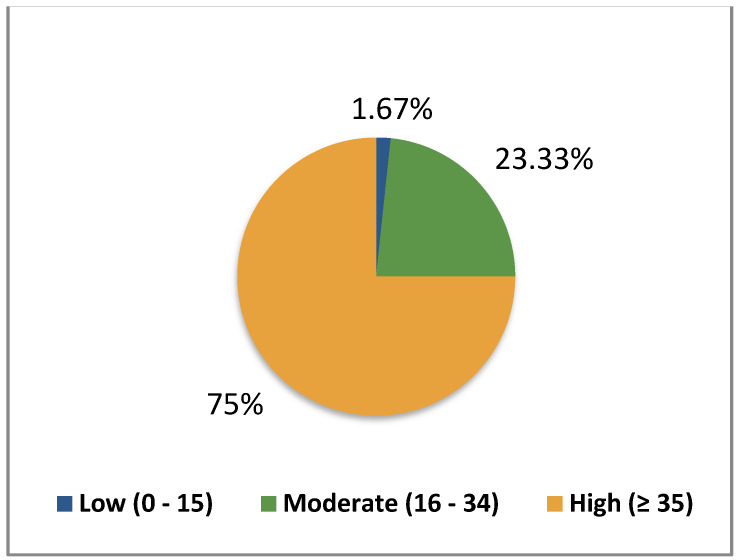
The Global Distress Scale cohorts based on the total sum of the answers to ESAS (%).

Most patients reported experiencing no pain upon admission (n = 22; 36.7%) or indicated severe pain, scoring greater than 7 on a scale from 0 to 10 (n = 19; 31.7%) (Table 3 and Figure 1).

**Figure 2:**
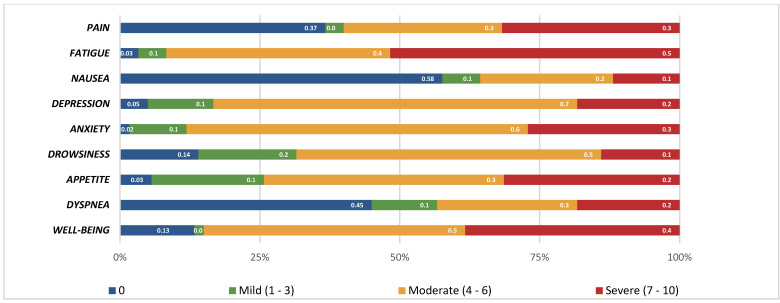
Frequencies of the patients’ answers to the ESAS (%) according to severity.

The majority of patients reported feelings of fatigue (n = 31; 51.7%), with scores exceeding 7 out of 10 (Table 3 and Figure 2). Only a few patients did not report fatigue (n = 2; 3.3%). More than half of the patients denied experiencing nausea (n = 34; 57.6%), with a mean nausea score of 2.30 (± 1.84). 

Depression was absent in a minority of patients (n = 3; 5%), while some patients reported a score of 6 out of 10 for depression (n = 19; 31.7%) (Table 3).

Drowsiness was absent in a small number of patients (n = 8; 14%). Conversely, the majority of patients reported a score higher than 5 out of 10 for drowsiness (n = 39; 68.4%) (Table 3). 

Loss of appetite was prevalent in most of the sample (n = 58; 96.6%), with some patients reporting a score of 5 out of 10 (n = 16; 26.7%) (Table 3). 

A significant number of patients (n = 27; 45%) denied experiencing dyspnea upon admission, while a few patients reported a score of 5 out of 10 (n = 10; 16.7%) (Table 3). 

Regarding overall well-being, some patients (n = 8; 13.35%) scored 0 on well-being (no well-being) and others (n = 20; 33.3%) reported 6/10, with a mean of 5.55 (+/−2.37), indicating a moderate presence of well-being in this sample.

### 3.3. Palliative Outcome Scale (POS) Results

The scores from the Palliative Outcome Scale (POS) are presented in Table 4 and Table 5, where the main topic of each question is identified. 

In this study, the question “If so, what were your main problems in the last 3 days?” was not included in the analysis. 

It was found that some patients felt that pain had a minimal impact on their daily lives (n = 20; 33.3%). However, the majority of patients (n = 25; 41.6%) reported that pain significantly impacted their daily activities.

For other symptoms, such as nausea, coughing, or constipation, there was a predominance of moderate (n = 19; 31.7%) and severe (n = 29; 48.3%) impacts.

Most patients reported feeling anxious and worried about disease progress and treatment. Specifically, some patients (n = 13; 21.7%) responded with “Sometimes—it affects my concentration now and then,” while others (n = 36; 60%) indicated “Most of the time—it often affects my concentration.”

Regarding the worry and anxiety of family members or friends, no patients selected the response “No, not at all.” Most patients perceived that their families were anxious and worried about them (n = 43; 72.9%).

Only a few patients reported having complete information or as much information as they desired (n = 9; 15.3%). Conversely, some patients (n = 20; 33.9%) acknowledged having access to information but faced difficulties in understanding it. The majority of patients expressed a desire for more information (n = 27; 45.8%), while a minority of the sample population indicated that they had no information about their disease (n = 3; 5.1%).

Additionally, it was found that most patients believed they were able to share their feelings most of the time or sometimes (n = 50; 84.8%).

Most patients felt that their lives were worthwhile, with 38 patients (63.3%) responding “Most of the time” and 13 patients (21.7%) indicating “Sometimes.” No patients reported that their lives were not worthwhile. Additionally, the majority of patients felt good about themselves as individuals (n = 51; 85%), and no patients expressed negative feelings about themselves. When asked about wasted time, most patients responded with “None at all” (n = 50; 83.3%). A minority of patients reported “Up to half a day wasted” (n = 9; 15%) and “More than half a day wasted” (n = 1; 1.7%). Regarding whether any practical matters resulting from their illness, either financial or personal, had been addressed, most patients (n = 41; 68.3%) stated, “Practical problems have been addressed, and my affairs are as up to date as I would wish.” Some patients (n = 11; 18.3%) reported that “Practical problems are in the process of being addressed,” while others (n = 8; 13.3%) indicated “I have had no practical problems.”

As shown in Table 5, the majority of patients (n = 49; 81.7%) completed this questionnaire independently. A few patients received assistance from family members or friends (n = 6; 10%) and from palliative care staff (n = 5; 8.3%).

### 3.4. ESAS and POS Measures According to the Sociodemographic Characteristics, Type of Cancer and ECOG

In Table 6, the authors present a univariate analysis for the total POS and ESAS score with clinical and social factors. 

There was no clinical or social factor associated with total ESAS and POS score, except the number of children, which was significantly related with POS score. 

**Figure 3:**
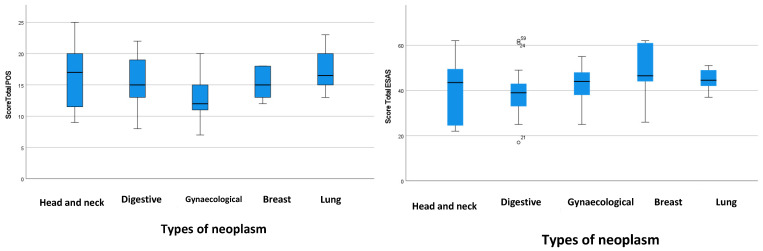
Distribution of ESAS-GDS and POS total score according to the types of neoplasm.

The total scores of the ESAS-GDS and POS according to the type of cancer are present in Figure 3. 

Patients with head and neck tumors exhibited a median Palliative Outcome Scale (POS) score of 17 [13; 19] and a median Edmonton Symptom Assessment System—Global Distress Scale (ESAS-GDS) score of 43.5 [34.25; 48]. In contrast, patients with digestive cancer reported a median ESAS score of 39 [34.25; 48] and a median POS score of 15 [13; 19].

Patients with breast and gynecological tumors demonstrated very similar results regarding median Edmonton Symptom Assessment System—Global Distress Scale (ESAS-GDS) and Palliative Outcome Scale (POS) scores. In breast cancer patients, the median POS score was 15 [13; 18.75], while the median ESAS score was 46.5 [34.25; 48]. For patients with gynecological tumors, the median POS score was 12 [12.5; 19] and the median ESAS score was 44 [36; 48.5].

Patients with lung cancer presented a median POS score of 16.5 [13; 19] and median ESAS score of 44.5 [35.5; 48]. 

**Figure 4:**
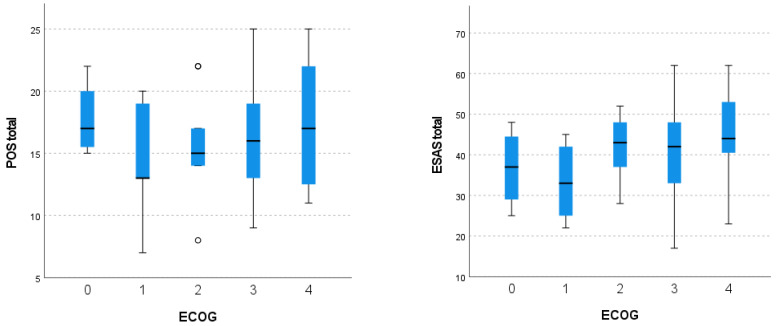
Distribution of ESAS-GDS and POS total score according to the ECOG status.

The total scores of the ESAS-GDS and POS according to the ECOG status are present in Figure 4.

Patients who were fully active (ECOG 0) exhibited a median POS score of 17.75 [15.25; 21] and a median Edmonton Symptom Assessment System–Global Distress Scale (ESAS-GDS) score of 36.75 [27; 46.25].

On the other hand, patients with some restrictions in physically strenuous activities (ECOG 1) reported a median ESAS score of 33.3 [24.25; 42.75] and a median POS score of 14.17 [11.5; 19.25]. 

Patients that were capable of all self care but unable to carry out work activities (ECOG 2) presented a median ESAS score of 42.10 [36.5; 48.75] and median POS score of 15.70 [14; 18.25]. 

Furthermore, patients that were only capable of limited self care and were confined to a bed or chair for more than 50% of their waking hours (ECOG 3) demonstrated a median POS score of 16.16 [13; 19] and ESAS score of 40.68 [33; 48]. Patients completely disabled and totally dependent on others (ECOG 4) presented very similar results regarding ESAS (46.53 [39; 55]) and POS score (16.8 [12; 22]). 

**Figure 5:**
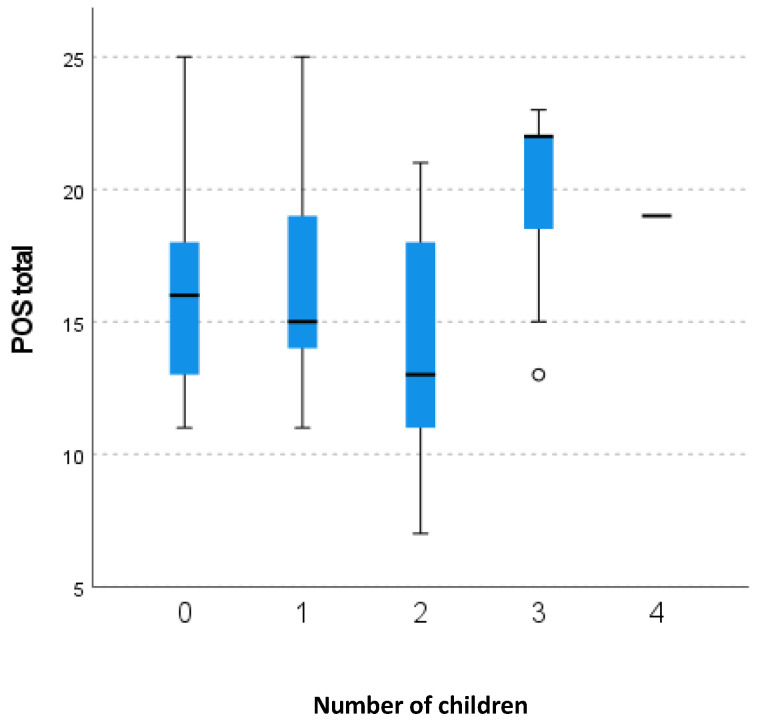
Distribution of POS total score according to the number of children.

In Figure 5, the authors demonstrate the analysis between total POS score and the number of children of the patients observed. This analysis is statistically significant. 

Patients without children or with only one child exhibited a median POS score of 16 [11; 18] and 16.71 [13.5; 19.50], respectively. On the other hand, patients with two children presented a median POS score of 14 [11; 18]. Finally, patients with three or more children showed a median POS score of 19.86 [15; 22]. 

There was no significant correlation between age with ESAS (r = −0.069, *p* = 0601) and POS (r = 0.099, *p* = 0.452) score. 

### 3.5. Correlation Between ESAS and POS

An analysis was conducted to evaluate the correlation between symptom severity, as measured by the ESAS-GDS, and quality of life, as assessed by the POS. 

The results demonstrated a positive and statistically significant correlation between the total scores of the POS and ESAS-GDS scales (*p* < 0.01). This finding indicates that higher GDS scores, which reflect poorer symptom control, are associated with higher POS scores, signifying a deterioration in quality of life (Table 7).

Furthermore, a significant correlation was identified between pain, depression, anxiety, and loss of appetite, all with *p* < 0.01, and quality of life. A similar positive correlation was observed between fatigue and quality of life, albeit with *p* < 0.05. Thus, the high severity of these symptoms is associated with a decline in quality of life.

## 4. Discussion

In this specific population of hospitalized cancer patients, there was a predominance of the male gender, with a median age of 72 years (range: 43–94), which differs significantly from other studies. For instance, Karaman et al. conducted a study evaluating the Edmonton Symptom Assessment Scale (ESAS) in a palliative care population, reporting a mean age of 54.04 ± 15.89 years and a predominance of the female gender (51.9%) [30]. Antunes and Ferreira conducted a study to validate the Integrated Palliative Care Outcome Scale (IPOS) in a Portuguese palliative population, which revealed a mean age of 66.8 ± 12.7 years [31]. Kaasa et al. conducted a survey examining patient demographics in European palliative care units, which revealed a mean age of 66 years and a predominance of the female gender (56%) [32]. 

This difference can be attributed to the types of cancer observed in the study. A notable predominance of digestive cancers (41.7%) and head and neck cancers (20%) was found. Digestive system cancers are among the most lethal for men, who also exhibit a higher susceptibility to head and neck cancers [33,34]. In 2019, data indicated that head and neck cancers were 1.2 times more prevalent in males than in females [33,34].

Modonesi et al. conducted a study in an Italian palliative care unit, which yielded somewhat different results. In their study, the most frequently encountered cancer site was digestive tumors (30.2%), followed by lung tumors (18.5%) and breast tumors (11.1%) [35]. The introduction of immunotherapy for lung cancer treatment, which has significantly improved survival rates, may account for its lower prevalence in our study. Since the approval of immune checkpoint inhibitors, the treatment paradigm for lung cancer and its survival outcomes have been markedly altered [36]. Studies have demonstrated a substantial improvement in survival rates among patients following the implementation of immunotherapy [37].

In this study, when evaluating the total ESAS and POS scores across the various tumor types, there are minimal differences observed. Patients with head and neck tumors exhibit a higher median POS score. Conversely, patients with breast, gynecological, and lung cancers demonstrate a higher median ESAS score.

We also presented an analysis of the total POS and ESAS score with ECOG and social factors. There was no clinical or social factor associated with total ESAS and POS score, except the number of children, which was significantly related with POS score.

The authors did not find data in the literature that would justify these results. However, we know that family caregivers play a key role in palliative care, and understanding the relationship between patient and family caregivers is important [38]. Family involvement, knowledge about disease and treatments, abilities to communicate with the patient and team improve caregiver and patients’ quality of life [39]. 

Families of palliative patients face various stressors, ranging from critical decisions about care to grief [40]. As they are faced with adverse circumstances, the different members of the family can be important resources, particularly for the patient [40]. However, these same circumstances could induce conflicts [40]. The types of conflicts range from different needs or preferences for communication [40]. Disputes may arise due to the perception of insufficient assistance from a particular family member [41], the difficulty of reaching agreement on the coordination of patient care and reappearance of past tensions [42]. From the authors’ perception, these conflicts can be accentuated in larger families, with members with different coping styles. Family conflicts in themselves can be a source of stress for the patient and affect their quality of life. 

The Edmonton Symptom Assessment System (ESAS) is a credible and validated instrument for assessing the intensity of symptoms in palliative care, facilitating their identification and monitoring [10]. Over time, the management of pain and nausea has improved significantly through the use of targeted and effective therapies [43]. However, other symptoms, such as anorexia and the overall sense of well-being, have received comparatively less attention in research [43]. Current treatments for these symptoms have demonstrated limited effectiveness, raising important questions about the true impact of palliative care in alleviating such symptoms and the associated suffering [43].

In this study, symptoms such as pain, nausea, drowsiness, and dyspnea were reported with mild to moderate scores, likely reflecting an earlier initiation of palliative care assessments in both internal and external consultations. According to Cochrane reviews, early palliative care interventions can yield more beneficial effects not only in symptom intensity and management but also in overall quality of life [44]. While the authors noted only small effect sizes, these may still be clinically significant in advanced disease contexts where prognosis is limited [44]. 

Symptoms such as fatigue, depression, anxiety, loss of appetite, and well-being received the highest scores in this sample, indicating that these symptoms were more challenging to manage. Loss of appetite and weight loss are prevalent in advanced cancer cases [45]. However, cachexia cannot be solely attributed to inadequate caloric intake, as it is not reversed by caloric supplementation [45]. Biochemical alterations, including anemia, inflammation, and low albumin levels, play significant roles in this process [45,46].

Anorexia is one of the most common symptoms encountered in palliative care [46]. In a study involving a sample of 3030 patients receiving palliative care, 26% reported experiencing moderate or severe anorexia [47]. The pharmacologic treatments currently available predominantly include appetite stimulants; however, these interventions generally do not reverse cachexia in most patients [48]. 

Similarly to anorexia, many patients in palliative care experience fatigue [49]. Due to its multifactorial etiology, the assessment and treatment of fatigue can be challenging [49]. It is often necessary to investigate potential reversible causes that may be amenable to treatment [49]. If such causes are not identified, there are limited pharmacological options available for patients in palliative care [49]. Additionally, non-pharmacological approaches have shown limited efficacy [49].

Non-pharmacological therapies constitute complementary treatments in the area of palliative care, which aim to improve patients’ quality of life, reducing symptom burden [43]. In the literature, some of the non-pharmacological interventions with an impact on oncological palliative care include physiotherapy, music therapy, massage therapy, cognitive–behavioral strategies and aromatherapy [50]. In particular for fatigue, aerobic exercise has consistent effects on depression and sleep quality [51].

In the case of anorexia, non-pharmacological interventions include dietary advice and assistance in reducing food-related stress, particularly in relation to family members [52]. The promotion of small, energy-rich and appetizing-looking meals constitutes an effective type of non-pharmacological intervention in daily clinical practice in this area, reducing patient and family stress in relation to meals [53]. It should be noted that some studies have shown that patients who lived alone had more loss of appetite upon admission, with improvement during hospitalization [54]. The social aspect of eating is crucial in treating loss of appetite [54]. Assistance with food preparation and initiatives such as shared meals with other patients, caregivers and/or healthcare professionals can be beneficial during hospitalization [54].

Life-threatening illnesses can be significant triggers for anxiety and depression [45]. In addition to addressing physical needs, it is crucial to consider psychological, social, and spiritual needs as well [45]. Care teams must prioritize all these dimensions when providing care to patients in palliative settings and should be equipped with the multidisciplinary human resources necessary to address them effectively [45].

The Palliative Outcome Scale (POS) is a psychometrically reliable tool designed to assess the quality of life of patients in palliative care [21]. It effectively identifies five key areas: emotional well-being, consequences of the disease on daily life, received information and support, anxiety, and burden of disease [12].

It was observed that symptoms such as nausea, coughing, and constipation, as well as anxiety experienced by both patients and their families, significantly impacted the quality of life in our sample. The healthcare team should prioritize early identification and management of both physical and emotional symptoms. Siemens et al. found that approximately 20% of patients newly diagnosed with advanced oncological disease exhibited considerable levels of anxiety and depression, which were associated with a significant decline in quality of life [45]. Identifying previously unrecognized palliative needs is essential for achieving better outcomes [49]. Without adequate symptom screening, patients may experience diminished well-being, negatively affecting their daily activities, sleep quality, and interpersonal relationships [55].

Finally, the authors would like to highlight that this study was carried out in patients with palliative needs, in the context of oncological disease. However, we know that several non-oncological diseases are also indicated for palliative care. Indeed, symptomatic control in patients in these two contexts may be different, because the symptom burden may differ [56]. Patients with non-oncological diseases had shorter episode durations and were more affected by symptom clusters [56]. On the other hand, oncological patients had an additional communicational/practical cluster [56]. Non-oncological patients had significantly higher odds of fatigue, insomnia and impairment in emotional and physical functioning, but also in quality of life, while cancer patients had higher odds of pain [57].

### Limitations

This study has several limitations that warrant mention.

First, the sample size was relatively small, which was influenced by the research being conducted during the COVID-19 pandemic. This period significantly impacted patients’ access to palliative care, particularly concerning the assessment of palliative needs, restrictions on visitation, and the increase in telemedicine consultations [58].

Additionally, conducting this investigation within a single hospital further constrained the sample size. Future studies should aim to include more palliative care units and a larger and more diverse patient population in order to reduce bias and give a more insight into demographic and symptomatic trends in Portuguese palliative care.

Another limitation was the exclusion criteria. The authors excluded all patients who did not speak Portuguese, had communication difficulties, were terminally ill or were unable to comprehend the study and/or provide written consent.

Including patients with communication difficulties or cognitive impairment, potentially with caregiver reporting, could capture data from a broader spectrum of palliative populations. On the other hand, addressing linguistic diversity by including Portuguese interpreters or translated material would also increase sample inclusion. 

Finally, the cross-sectional design of the study, in which the researchers contacted the study participants just once, allowed us to understand the current status of the patients but did not enable us to evaluate the continuity of care and the impact of care on the control and management of symptoms of people with palliative needs. Therefore, a longitudinal study is recommended for future investigations.

## 5. Conclusions

This study aims to provide a comprehensive profile of symptomatic intensity and patients’ perceptions of quality of life among Portuguese oncological palliative patients at the time of hospital admission.

Our findings indicate a positive correlation between high severity of global symptoms and a perception of reduced quality of life. When analyzing specific symptoms, we observed a positive correlation between poor quality of life and symptoms such as pain, fatigue, depression, anxiety, and anorexia/loss of appetite. Uncontrolled pain and depression have negative consequences on the quality of life of these patients and their caregivers. It is known that optimizing symptomatic control increases the survival of patients in palliative care [59,60,61].

Conversely, it was found that symptoms, along with self- and family-related anxiety, were the most significant issues impacting the quality of life in this population.

By incorporating both scales in this study, the authors were able to achieve a more comprehensive understanding of the physical and psychological symptoms experienced by patients with advanced cancer. It would be beneficial for future research to also include patients in palliative care for non-oncological conditions. A positive correlation was identified between the Palliative Outcome Scale (POS) and the Edmonton Symptom Assessment System–Global Distress Scale (ESAS-GDS), indicating that inadequate symptom control adversely affects the quality of life of these patients.

Future studies would benefit from a larger and more inclusive sample and also a longitudinal design to capture a broader range of palliative care experiences. Furthermore, the inclusion of additional measurement instruments to further assess symptom burden and quality of life would benefit palliative care investigations in Portugal. It would be effective to conduct the assessments earlier in the course of patients’ care to ensure symptom control and optimize quality of life throughout the disease.

## Figures and Tables

**Table 1:** Sociodemographic characteristics of the sample (n = 60).

Variables		Sample
Gender, n (%)	Male	37 (61.7)
	Female	23 (38.3)
Age (years)	Median [Q1; Q3]	72 [58, 25; 79]
	Minimum	43
	Maximum	94
Education level, n (%)	Elementary school	49 (81.7)
	High school	5 (8.3)
	Graduation	4 (6.7)
	Absent	2 (3.3)
Marital status, n (%)	Married	49
	Single	9 (15)
	Divorced	4 (6.6)
	Widow	16 (26.7)
Number of children, n (%)	0	13 (21.7)
	1	21 (35)
	2	18 (30)
	≥3	8 (13.3)
Residence, n (%)	Home	50 (83.3)
	Nursing home	10 (16.7)
Companions, n (%)	Spouse	30 (50)
	Son/daughter	7 (11.7)
	Siblings	1 (1.6)
	Father/mather/parents	3 (5)
	Without companion	12 (20)
	Other caregivers (eg nursing home)	7 (11.7)

Q1—first quartile; Q3—third quartile.

**Table 2:** Disease-related data and POS and ESAS scores of the sample (n = 60).

Variables		Sample
Origin service prior to admission, n (%)	Ambulatory/outpatient	34 (56.7)
	Hospitalization	26 (43.3)
Type of neoplasm, n (%)	Head and neck	12 (20)
	Cutaneous	3 (5)
	Digestive	25 (41.7)
	Gynecological	5 (8.3)
	Hematologic	1 (1.7)
	Breast	6 (10)
	Prostate	2 (3.3)
	Lung	6 (10)
POS Score *	Median [Q1; Q3]	15 [13; 19]
	Minimum	7
	Maximum	25
ESAS Score (GDS) *	Median [Q1; Q3]	42.5 [34.25; 48]
	Minimum	15
	Maximum	62
ECOG performance status at admission	0	4 (6.6%)
	1	6 (10%)
	2	10 (16.6%)
	3	25 (41.6%)
	4	15 (25%)

Q1—first quartile; Q3—third quartile; * Total score was obtained by adding the items.

**Table 3:** Frequencies of the patients’ answers to the ESAS (N (%)).

	ESAS										
	0	1	2	3	4	5	6	7	8	9	10
Pain	22 (36.7)	1 (1.7)	0 (0)	1 (1.7)	4 (6.7)	6 (10)	7 (11.7)	14 (23.3)	4 (6.7)	1 (1.7)	0 (0)
Fatigue	2 (3.3)	0 (0)	1 (1.7)	2 (3.3)	2 (3.3)	16 (26.7)	6 (10)	18 (30)	12 (20)	1 (1.7)	0 (0)
Nausea *	34 (57.6)	0 (0)	0 (0)	4 (6.8)	2 (3.4)	6 (10.2)	6 (10.2)	6 (10.2)	1 (1.7)	0 (0)	0 (0)
Depression	3 (5)	0 (0)	1 (1.7)	6 (10)	8 (13.3)	12 (20)	19 (31.7)	7 (11.7)	3 (5)	1 (1.7)	0 (0)
Anxiety *	1 (1.7)	0 (0)	0 (0)	6 (10.2)	3 (5.1)	14 (23.7)	19 (32.2)	10 (16.9)	5 (8.5)	1 (1.7)	0 (0)
Drowsiness **	8 (14)	1 (1.8)	4 (7)	5 (8.8)	0 (0)	24 (42.1)	7 (12.3)	1 (1.8)	5 (8.8)	2 (3.5)	0 (0)
Appetite	2 (3.3)	1 (1.7)	1 (1.7)	3 (5)	5 (8.3)	16 (26.7)	7 (11.7)	11 (18.3)	9 (15)	5 (8.3)	0 (0)
Dyspnea	27 (45)	0 (0)	2 (3.3)	5 (8.3)	2 (3.3)	10 (16.7)	3 (5)	6 (10)	2 (3.3)	3 (5)	0 (0)
Well-being	8 (13.3)	0 (0)	0 (0)	1 (1.7)	0 (0)	8 (13.3)	20 (33.3)	15 (25)	7 (11.7)	1 (1.7)	0 (0)

* 1 missing; ** 3 missing.

**Table 4:** Frequencies of the patients’ answers to the first 10 questions of the POS (N (%)).

POS					
*Items/Score*	0	1	2	3	4
1. Have you been affected by PAIN?	20 (33.3)	4 (6.7)	11 (18.3)	23 (38.3)	2 (3.3)
2. Have OTHER SYMPTOMS (nausea, cough, constipation) seemed to be affecting how you feel?	8 (13.3)	2 (3.3)	19 (31.7)	29 (48.3)	2 (3.3)
3. Have you been feeling ANXIETY ABOUT DISEASE or treatment?	1 (1.7)	6 (10)	13 (21.7)	36 (60)	4 (6.7)
4. Have any of your FAMILY been ANXIOUS about you? *	0 (0)	2 (3.4)	12 (20.3)	43 (72.9)	2 (3.4)
5. How much INFORMATION have you and your family been given? *	9 (15.3)	20 (33.9)	27 (45.8)	1 (1.7)	2 (3.4)
6. Have you been able to SHARE how you are feeling? *	5 (8.5)	29 (49.2)	21 (35.6)	4 (6.8)	0 (0)
7. Have you felt that life was worthwhile (MEANING OF LIFE)?	7 (11.7)	38 (63.3)	13 (21.7)	2 (3.3)	0 (0)
8. Have you felt good about yourself as a person (SELF-FEELINGS)?	7 (11.7)	35 (58.3)	16 (26.7)	2 (3.3)	0 (0)
9. How much TIME do you feel has been wasted on appointments relating to your healthcare (LOST TIME)?	50 (83.3)	-	9 (15)	-	1 (1.7)
10. Have any PRACTICAL matters resulting from your disease, either financial or personal, been addressed?	41 (68.3)	-	11 (18.3)	-	8 (13.3)

* 1 missing. Question 1, 2: 0—no; 1—slightly; 2—moderately; 3—severely; 4—overwhelmingly. Question 3, 4: 0—no; 1—Occasionally; 2—Sometimes; 3—Most of the time; 4—always. Question 5: 0—Full information or as much as wanted; 1: Information given but hard to understand; 2—Information given on request but would have liked more; 3—Very little given and some questions were avoided; 4—None at all. Question 6, 7, 8: 0—always; 1—Most of the time; 2—Sometimes; 3—Occasionally; 4—never. Question 9: 0—None at all; 2—Up to half a day wasted; 4—More than half a day wasted. Question 10: 0—I have had no practical problems; 2—Practical problems are in the process of being addressed; 4—Practical problems exist which were not addressed.

**Table 5:** Frequencies of the patients’ answers to the last question of the POS, which does not enter in the final score (N (%)).

	Alone (0)	With Help of Family or Friend (1)	With Help of Palliative Care Staff (2)
How did you complete this questionnaire?	49 (81.7)	6 (10)	5 (8.3)

**Table 6:** Univariate analysis for the total POS and ESAS overall score.

Variable	ESAS-GDS Total Score *		POS Total Score *	
	*Effect (chi)*	*p Value*	*Effect (chi)*	*p Value*
**Gender**	4.849	0.089	2.396	0.302
**Marital status**	4.541	0.474	2.462	0.782
**Education level**	6.369	0.383	7.600	0.269
**Number of children**	6.510	0.164	10.420	0.034 **
**Companion**	2.626	0.757	3.815	0.576
**Residence**	0.819	0.845	3.885	0.274
**Types of neoplasm**	7.262	0.402	5.493	0.6
**ECOG**	7.602	0.107	1.827	0.768

ESAS—Edmonton Symptom Assessment Scale; ECOG—Eastern Cooperative Oncology Group. * Analyses performed with Kruskal–Wallis; ** *p* < 0.05.

**Table 7:** Spearman’s correlations between the total POS score and the ESAS (total and each item).

	Total POS Score
Pain	0.356 **
Fatigue	0.270 *
Nausea	0.090
Depression	0.382 **
Anxiety	0.415 **
Drowsiness	0.228
Appetite	0.352 **
Dyspnea	0.077
Well-being	0.171
Total ESAS Score	0.448 **

* *p* < 0.05; ** *p* < 0.01.

## Data Availability

The datasets generated and analyzed during the current study are available from the corresponding author upon reasonable request.
